# Exceptional Response to Pembrolizumab Immunotherapy in a Young Patient With Lynch Syndrome-Associated Metastatic Colorectal Cancer

**DOI:** 10.7759/cureus.107078

**Published:** 2026-04-15

**Authors:** Muhammad Niazi, Richard Crossley

**Affiliations:** 1 Department of Medical Oncology, Weston Park Cancer Centre, Sheffield, GBR; 2 Department of Clinical Oncology, Weston Park Cancer Centre, Sheffield, GBR

**Keywords:** checkpoint inhibitor, colorectal cancer, complete pathological response, immunotherapy, lynch syndrome, mismatch repair deficiency, msi-h, pembrolizumab

## Abstract

Microsatellite instability-high (MSI-H) colorectal cancers demonstrate significant responses to immune checkpoint inhibitors. Lynch syndrome, caused by germline mismatch repair gene mutations, typically presents with MSI-H tumours. We report a remarkable case of complete pathological response to pembrolizumab in a young patient with extensive Lynch syndrome-associated metastatic colorectal cancer. A woman in her early 30s with a strong family history of malignancy presented with progressive fatigue and anaemia. Investigations revealed moderately differentiated adenocarcinoma of the hepatic flexure with extensive metastatic disease, including innumerable liver metastases, retroperitoneal lymphadenopathy, and ascites. Molecular testing demonstrated MSI-H status with loss of MutS Homolog 2 (MSH2) and MutS Homolog 6 (MSH6) expression, v-Raf murine sarcoma viral oncogene homolog B (BRAF) wild-type, consistent with Lynch syndrome. Baseline carcinoembryonic antigen (CEA) was approximately 272 ng/mL. In a healthy adult who smokes, CEA is considered within normal limits at a level less than or equal to 5 ng/ml. Given the MSI-H phenotype, first-line pembrolizumab immunotherapy was commenced rather than conventional chemotherapy. The patient demonstrated a dramatic, sustained response over 24 months of pembrolizumab. CEA declined from approximately 300 ng/mL to 1.0 ng/mL. Serial imaging showed progressive tumour reduction with transformation of solid liver metastases into cystic lesions. Post-treatment Positron Emission Tomography-Computed Tomography (PET-CT) demonstrated a complete metabolic response. Following the completion of immunotherapy, the patient underwent right hemicolectomy. Histopathological examination revealed no viable cancer cells in the entire surgical specimen, confirming complete pathological response (ypT0, ypN0, R0). Immune-related adverse events were manageable and did not require treatment discontinuation. The patient remains under active surveillance with stable residual cystic liver lesions and no evidence of disease progression. This case demonstrates that complete pathological responses to pembrolizumab are achievable in MSI-H metastatic colorectal cancer. Universal MSI/mismatch repair (MMR) testing is essential to identify candidates for first-line immunotherapy, and Lynch syndrome should be considered in young patients with MSI-H tumours and a family history of malignancy.

## Introduction

Colorectal cancer represents a major global health burden, with approximately 25% of patients presenting with metastatic disease at diagnosis [1]. Lynch syndrome, a hereditary colorectal cancer syndrome caused by germline mutations in mismatch repair (MMR) genes, accounts for 2-5% of all colorectal cancers and is associated with an increased risk of gynaecological, urinary tract, and other gastrointestinal malignancies [2].

Microsatellite instability-high (MSI-H) status, resulting from deficient mismatch repair (dMMR), occurs in approximately 15% of early-stage colorectal cancers but in only 4-5% of metastatic cases. The landmark Phase 3, open-label, randomized study of first-line pembrolizumab versus investigator-choice chemotherapy for dMMR or MSI-H metastatic colorectal carcinoma (KEYNOTE-177) trial established pembrolizumab as first-line therapy for MSI-H/dMMR metastatic colorectal cancer, demonstrating superior progression-free survival (16.5 vs 8.2 months) and overall response rates (43.8% vs 33.1%) compared to chemotherapy, with approximately 11% of patients achieving a complete radiographic response [3].

Complete pathological responses - defined as the absence of any viable tumour cells in the resected surgical specimen - represent the optimal outcome of immunotherapy and are considerably rarer than complete radiographic responses [3]. This case demonstrates such an exceptional outcome in a young patient with Lynch syndrome-associated extensive metastatic disease, and highlights critical aspects of molecular profiling, immunotherapy efficacy, adverse event management, and hereditary cancer syndrome surveillance.

## Case presentation

History

A woman in her early 30s presented to her general practitioner in September 2022 with progressive fatigue, initially attributed to a pre-existing history of chronic fatigue syndrome following severe Epstein-Barr virus infection five years prior. She had a significant family history of malignancy: her mother died in her early-to-mid 50s from recurrent cervical cancer, a maternal aunt developed gastric cancer, and her maternal grandmother died from cancer in her 90s. Her personal medical history included cervical intraepithelial neoplasia grade 3 (CIN3). She was an ex-smoker with minimal alcohol consumption.

As symptoms progressed, she developed upper abdominal pain. Abdominal ultrasound revealed gallstones. Worsening anaemia subsequently necessitated blood transfusion and intravenous iron infusion. Faecal immunochemical testing (FIT) was positive (threshold ≥10 µg haemoglobin/g faeces per National Institute for Health and Care Excellence diagnostics guidance (NICE DG56 [4]), prompting urgent colorectal referral.

Examination

Physical examination at presentation was unremarkable aside from signs consistent with anaemia. The patient maintained performance status zero throughout her treatment course.

Investigations

**Figure 1:**
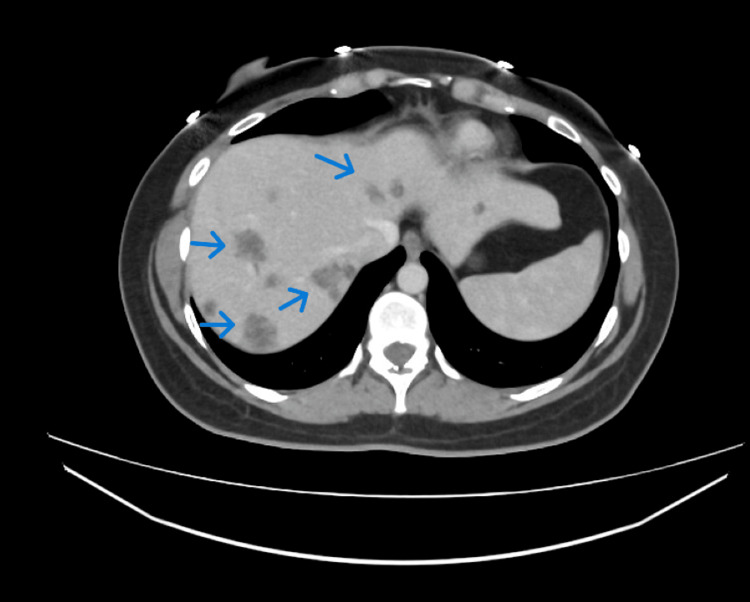
CT abdomen with contrast performed in December 2022 demonstrating multiple liver metastasis (blue arrow)

Colonoscopy in early December 2022 revealed a highly suspicious lesion at the transverse colon/hepatic flexure. Histopathology confirmed moderately differentiated adenocarcinoma. Staging computed tomography (CT) demonstrated malignant stricture of the proximal/mid transverse colon with direct extension into adjacent pericolic fat with innumerable bilobar liver metastases, retroperitoneal and portal lymphadenopathy, and ascites, consistent with extensive metastatic disease. The cancer was deemed incurable at presentation (Figure 1).

Molecular genetic testing of the tumour biopsy revealed MSI-H status with loss of MutS Homolog 2 (MSH2) and MutS Homolog 6 (MSH6) protein expression on immunohistochemistry and v-Raf murine sarcoma viral oncogene homolog B (BRAF) wild-type status. The baseline carcinoembryonic antigen (CEA) was 272 ng/mL.

The constellation of findings - MSI-H status, loss of MSH2 and MSH6, absence of BRAF mutation, young age at presentation, and strong family history of Lynch syndrome-associated cancers including cervical, gastric, and other malignancies - was highly consistent with Lynch syndrome. The patient was referred to the genetics service for formal assessment and counselling.

Differential diagnosis

The constellation of findings was highly suggestive of Lynch syndrome, which was subsequently confirmed on germline genetic testing. Sporadic MSI-H colorectal cancer was considered less likely given the patient's young age and family history, as sporadic MSI-H tumours typically occur in older patients and usually arise through MutL Homolog 1 (MLH1) promoter hypermethylation with concomitant BRAF mutation, neither of which were present. Constitutional mismatch repair deficiency (CMMRD) was considered unlikely given the age of presentation, as CMMRD typically presents in childhood or adolescence with multiple primary malignancies and features of neurofibromatosis type 1.

Systemic therapy

Following multidisciplinary team (MDT) discussion involving an oncologist, a radiologist, surgeons and a pathologist, the patient was offered first-line pembrolizumab immunotherapy rather than conventional FOLinic acid, Fluorouracil, Irinotecan (FOLFIRI) or FOLinic acid, Fluorouracil, OXaliplatin (FOLFOX) chemotherapy (with or without panitumumab), based on the MSI-H status. The treatment was palliative in intent given the extensive metastatic burden, though immunotherapy offered the possibility of superior disease control and quality of life compared to chemotherapy. Pembrolizumab 200 mg, given intravenously every three weeks, was commenced in January 2023. The dosing schedule was later transitioned to six-weekly administration in June 2023. Treatment continued for the full 24-month licensed duration, completing in December 2024.

Surgical management

Given the exceptional response to immunotherapy, the MDT discussed potential surgical options. Liver transplantation was considered; however, following referral to the liver transplant MDT, the patient was deemed unsuitable due to apparent lymph node involvement along the porta hepatis, which constitutes a contraindication. The transplant team also concluded that transplantation was probably unnecessary given the excellent response, with residual liver lesions appearing cystic and metabolically inactive on imaging.

Resection of the primary colorectal tumour was ultimately recommended for several reasons: the primary tumour site had developed a stricture not passable endoscopically, which limited the feasibility of future colonoscopic surveillance; Lynch syndrome surveillance requires regular colonoscopy for a minimum of 15-20 years; there was a high probability of no viable tumour remaining based on imaging response, with resection enabling definitive histopathological assessment; and the patient was young with excellent performance status and low surgical risk. Right hemicolectomy was performed in April 2025 without complications. Eighteen lymph nodes were harvested, all negative for metastasis, and histology confirmed complete resection at all margins.

Management of adverse events

In August 2023, thyroid dysfunction was identified with a thyroid-stimulating hormone level of 0.03 mIU/L (normal reference range 0.38-5.50 mIU/L). It was managed conservatively with repeat monitoring as per endocrinology advice. Thyroid dysfunction is a recognised immune-related adverse event of pembrolizumab, occurring in approximately 10-15% of patients [5]. Grade one immune-related hepatitis and pruritus were noted during the treatment course and resolved without the need for corticosteroids. The patient also experienced persistent dizziness and light-headedness. Notably, these symptoms predated her cancer diagnosis but appeared to worsen during immunotherapy. Extensive investigations including multiple brain MRI scans, CT head, comprehensive hormonal testing, ear, nose and throat (ENT) assessment, and neurology review revealed no definitive cause. The symptoms were managed symptomatically and did not require treatment discontinuation. The patient was referred to the chronic fatigue service given her pre-existing history.

Treatment response and imaging

**Figure 2:**
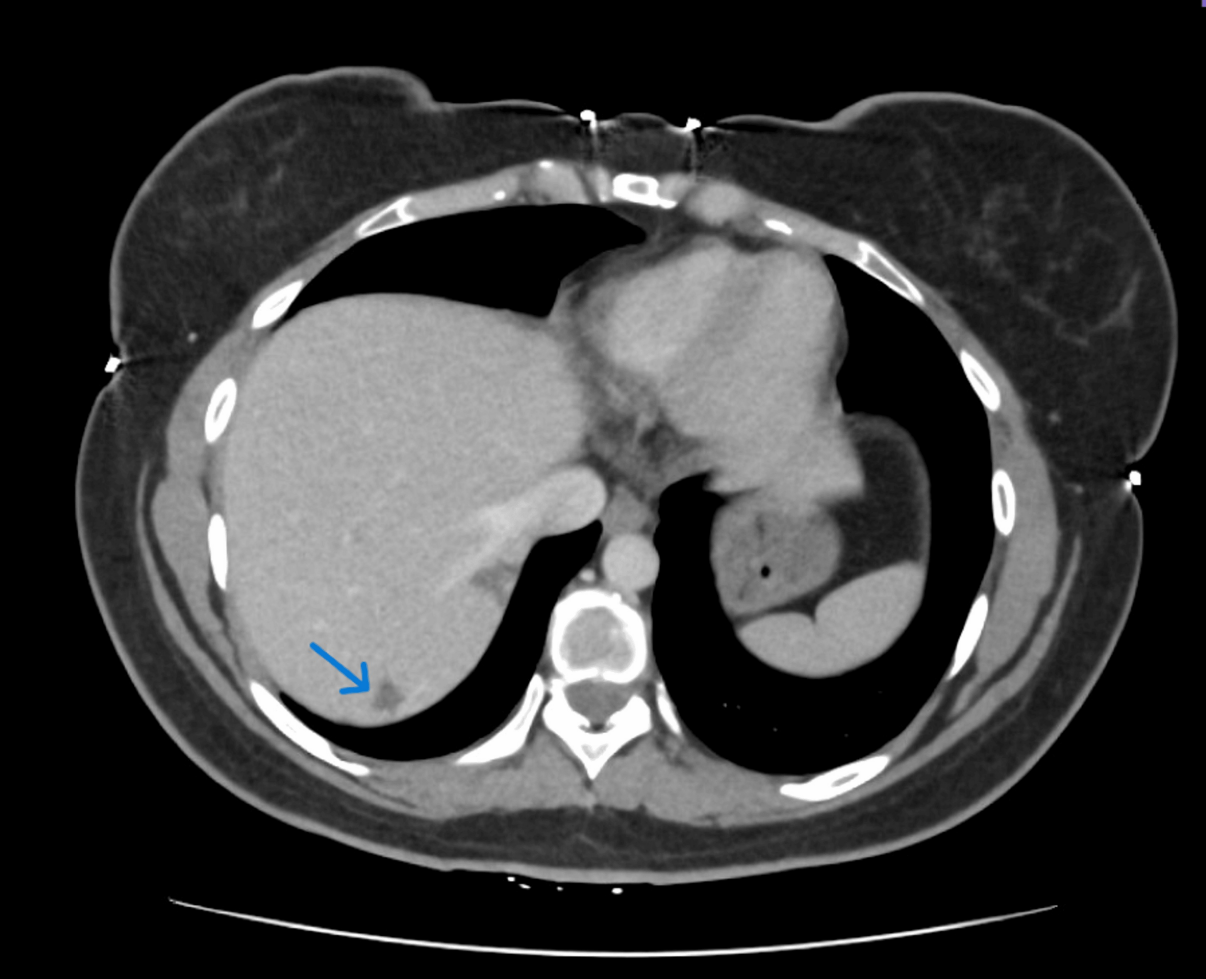
CT abdomen with contrast performed in December 2024 demonstrating cystic lesion in liver (blue arrow)

The patient demonstrated dramatic and sustained response to pembrolizumab over the 24-month treatment course. Baseline CEA was 272 ng/mL, ultimately reaching 1.0 ng/mL by completion in December 2024, representing an approximately 300-fold reduction. Serial CT imaging demonstrated progressive reduction in primary tumour size and transformation of solid liver metastases into cystic structures (Figure 2).

**Figure 3:**
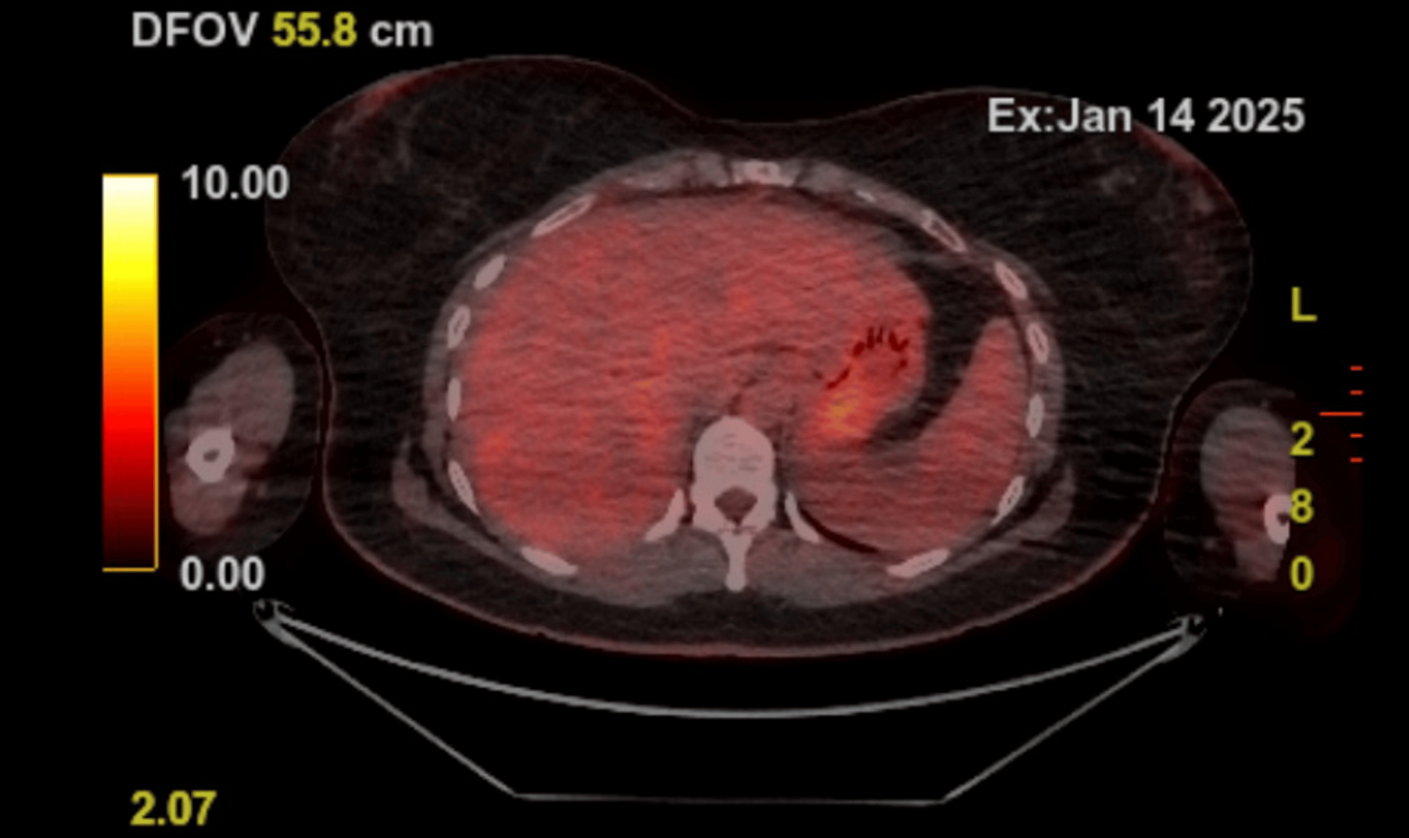
PET-CT scan performed in January 2025 demonstrating no metabolic activity outside normal physiological metabolism PET-CT: Positron Emission Tomography-Computed Tomography.

In February 2025, colonoscopy revealed a benign-appearing stricture at the primary tumour site; the scope was unable to pass the stricture. Post-treatment Positron Emission Tomography-Computed Tomography (PET-CT) demonstrated no metabolic activity outside normal physiological metabolism, confirming complete metabolic response (Figure 3).

Pathological findings

Histopathological examination of the right hemicolectomy specimen revealed remarkable findings. No viable cancer cells were identified in the entire resection specimen. The site of the original primary tumour showed fibrosis and tissue architecture changes consistent with treated malignancy, but no residual viable adenocarcinoma. All examined lymph nodes (0/18) were negative for malignancy. Staging was classified as ypT0, ypN0, R0, confirming a complete pathological response (pCR) to pembrolizumab immunotherapy.

Current status and ongoing surveillance

The patient remains under active surveillance following completion of the 24-month pembrolizumab course in December 2024 and surgical resection in April 2025. CEA remains below 1.0 ng/mL. Serial CT imaging continues to show stable residual cystic liver lesions with no evidence of new or progressive disease. The hepatobiliary team has confirmed these findings are consistent with treated disease and recommends ongoing surveillance imaging. The patient has been established with the genetics service for Lynch syndrome surveillance, incorporating regular colonoscopic surveillance (now facilitated by removal of the colonic stricture), endometrial surveillance with pelvic ultrasound, and surveillance for other Lynch syndrome-associated malignancies. Genetic counselling and cascade family testing have been arranged. The patient commenced aspirin 300 mg daily for Lynch syndrome-associated cancer risk reduction following the genetics team recommendation, in line with evidence from the Colorectal Adenoma/carcinoma Prevention Programme 2 (CAPP2) trial demonstrating a sustained reduction in colorectal cancer risk with aspirin in Lynch syndrome carriers [6]. She remains well with excellent performance status and has returned to normal daily activities.

Patient's perspective

Throughout her treatment journey, the patient demonstrated remarkable resilience and maintained an optimistic outlook. Despite the initial devastating diagnosis of widespread metastatic disease, she engaged actively with the treatment process and maintained excellent quality of life. The patient expressed gratitude for the opportunity to receive immunotherapy rather than chemotherapy, noting minimal treatment-related side effects and the ability to continue many normal daily activities during treatment. She is now focused on her ongoing surveillance programme.

## Discussion

This case demonstrates the transformative potential of immune checkpoint inhibition in MSI-H metastatic colorectal cancer, particularly in the context of Lynch syndrome. The patient achieved a pCR in the primary cancer specimen following pembrolizumab monotherapy, with long-term durability of response remaining to be determined during ongoing surveillance. We now have the nivolumab plus ipilimumab immunotherapy combination approved in the UK, based on the CheckMate 8HW phase 3 trial for patients with unresectable or metastatic colorectal cancer and microsatellite instability-high or mismatch repair-deficient status. This option of combination immunotherapy was not available as NICE approved the treatment in December 2022. Therefore, our patient was offered treatment with pembrolizumab immunotherapy instead. CheckMate 8HW compared treatment with nivolumab plus ipilimumab versus nivolumab and showed superior progression-free survival with the combination as compared to single-agent immunotherapy [7].

MSI-H colorectal cancer and immunotherapy

Microsatellite instability arises from deficient mismatch repair, resulting in high tumour mutational burden and neoantigen generation that render these cancers highly immunogenic [3]. The KEYNOTE-177 trial demonstrated that pembrolizumab significantly improved progression-free survival compared to chemotherapy in the first-line treatment of MSI-H/dMMR metastatic colorectal cancer [3]. While objective response rates were superior with pembrolizumab, complete responses remained uncommon at approximately 11% on radiographic assessment [3]. pCR with no viable tumour cells in primary cancer - as seen in our patient - are even rarer and represent the optimal outcome of immunotherapy.

Lynch syndrome considerations

The pattern of MSH2 and MSH6 protein loss, combined with absence of BRAF mutation, young age, and strong family history, was highly suggestive of Lynch syndrome from the outset. Several features were characteristic of this hereditary syndrome: young age at diagnosis, right-sided tumour location, MSI-H/dMMR phenotype, and family history including extracolonic malignancies such as cervical and gastric cancers, which are both recognised Lynch syndrome-associated cancers. The identification of Lynch syndrome carries important implications beyond the index patient, requiring genetic counselling, cascade testing of at-risk relatives, and lifelong surveillance for Lynch syndrome-associated cancers [2]. As per NHS England guidelines and the British Society of Gastroenterology, Association of Coloproctology of Great Britain and Ireland, and United Kingdom Cancer Genetics Group (BSG/ACPGBI/UKCGG) 2020 hereditary colorectal cancer guidelines, family members of index Lynch syndrome cases undergo cascade germline testing via regional genetics services and the National Disease Registration Service (NDRS) registry. Confirmed pathogenic variant carriers are enrolled into the NHS Lynch Syndrome Bowel Cancer Screening Programme (LS-BCSP, launched in 2023), offering biennial high-quality colonoscopy from age 25 (MutL Homolog 1 (MLH1)/MSH2/Epithelial Cell Adhesion Molecule (EPCAM) carriers) or age 35 (MSH6/Postmeiotic Segregation Increased 2 (PMS2) carriers).

As no proven effective gynaecological screening exists within NHS England, risk-reducing surgery (hysterectomy with bilateral salpingo-oophorectomy) is offered from approximately age 40, once childbearing is complete, with ongoing gynaecological risk management coordinated through clinical genetics services. Aspirin chemoprevention should be discussed with Lynch syndrome patients, as the CAPP2 randomised trial demonstrated a sustained reduction in colorectal cancer incidence with regular aspirin use, with benefits persisting at 10-year follow-up [6].

Role of surgery after immunotherapy

The role of surgery in patients achieving excellent radiological response to immunotherapy for metastatic colorectal cancer is evolving. In this case, several factors supported surgical resection of the primary site: the strictured primary tumour site limiting future colonoscopic surveillance, Lynch syndrome necessitating regular lifelong colonoscopy, young age with excellent performance status and low surgical risk, and the opportunity for definitive histopathological assessment of response. The finding of pCR in primary cancer specimen not only confirms immunotherapy efficacy but also provides important prognostic information.

## Conclusions

This case demonstrates that complete pathological responses to pembrolizumab are achievable in MSI-H metastatic colorectal cancer. Universal molecular testing for MSI/MMR status should be performed in all patients with metastatic colorectal cancer to identify those who would benefit from immunotherapy rather than chemotherapy. Young patients with MSI-H colorectal cancer, particularly with right-sided tumours and a relevant family history, should be evaluated for Lynch syndrome with appropriate genetic counselling and cascade family testing. Immune-related adverse events require vigilant monitoring but are generally manageable and should not prevent patients from accessing immunotherapy. MDT discussion is essential in complex cases, particularly when considering surgical interventions after systemic therapy, and Lynch syndrome diagnosis necessitates lifelong multi-cancer surveillance.

## References

[1] Rodriguez-Bigas MA, Lin EH, Crane CH (2003). Stage IV colorectal cancer. Holland-Frei Cancer Medicine. 6th edition.

[2] Grover S, Syngal S (2010). Risk assessment, genetic testing, and management of Lynch syndrome. J Natl Compr Canc Netw.

[3] André T, Shiu KK, Kim TW (2020). Pembrolizumab in microsatellite-instability-high advanced colorectal cancer. N Engl J Med.

[4] (2026). Quantitative faecal immunochemical testing to guide colorectal cancer pathway referral in primary care. https://www.nice.org.uk/guidance/htg690.

[5] de Filette J, Jansen Y, Schreuer M, Everaert H, Velkeniers B, Neyns B, Bravenboer B (2016). Incidence of thyroid-related adverse events in melanoma patients treated with pembrolizumab. J Clin Endocrinol Metab.

[6] Burn J, Sheth H, Elliott F (2020). Cancer prevention with aspirin in hereditary colorectal cancer (Lynch syndrome), 10-year follow-up and registry-based 20-year data in the CAPP2 study: a double-blind, randomised, placebo-controlled trial. Lancet.

[7] André T, Elez E, Lenz HJ (2025). Nivolumab plus ipilimumab versus nivolumab in microsatellite instability-high metastatic colorectal cancer (CheckMate 8HW): a randomised, open-label, phase 3 trial. Lancet.

